# Osteoporosis Prevention—A Worthy and Achievable Strategy

**DOI:** 10.3390/nu2101073

**Published:** 2010-10-20

**Authors:** Howard A. Morris

**Affiliations:** 1 School of Pharmacy and Medical Sciences, University of South Australia, Adelaide, South Australia 5001, Australia; Email: howard.morris@health.sa.gov.au; 2 Chemical Pathology Directorate, SA Pathology, Adelaide, South Australia 5000, Australia

This special issue of Nutrients records seven of the presentations made to the very successful meeting titled “Osteoporosis Prevention: A Workshop on Calcium, Vitamin D and other Nutritional Aspects” held in Adelaide, Australia on 5 and 6 March 2010 [1,2,3,4,5,6,7]. Seventy six delegates attended from across Australia and New Zealand to review the current evidence that dietary calcium intake, vitamin D status, other nutrients and exercise play a significant role in bone mineral homeostasis and act to prevent the development of osteoporosis. The Workshop promoted the concept that osteoporosis is a predictable and preventable disease and that significant benefit would be achieved to reduce the incidence of osteoporosis and the risk of fractures from nutrition and life style activities. Such an achievement will not only save considerable pain, suffering and morbidity but will also have a major financial benefit for the healthcare system for which the cost of treatment for osteoporotic fractures already amounts to billions of dollars. 

A concrete proposal arising from the Workshop was the adoption by way of signing a petition addressed to the Federal Australian Government stating the following:

***We recommend to the Screening Subcommittee of the Australian Population Health Development Principal Committee (APHDPC) of the Australian Health Minister's Advisory Council that all women should be screened for bone density at the menopause with the aim of reducing post-menopausal bone loss and age-related fractures by adopting lifestyle measures, particularly diet and exercise.***

The session of the Workshop devoted to discussion of this statement agreed that the strongest evidence that lifestyle measures could improve bone status and prevent fractures was in postmenopausal women, since this is the time at which bone loss starts. It was agreed that women with below average Bone Mineral Density at the hip or spine, as indicated by a T-score below zero, were the most likely to benefit from lifestyle advice to preserve bone and reduce the risk of fractures. Issues identified for further investigation included whether it could be beneficial to measure bone density again at 5 and/or 10 years later. The actual interventions to be recommended and the optimal practices to ensure the highest adoption rate of such lifestyle measures would need further research and review. 

Professor B E Christopher Nordin was a driving force for instigating and organizing the Workshop. Speakers acknowledged his major contribution to osteoporosis research over some 60 years. In particular he was recognized for his inspirational leadership for research and advocacy of the importance of calcium deficiency in the etiology of postmenopausal osteoporosis. His prodigious productivity as a medical researcher includes his outstanding publishing output of over 500 scientific papers and his achievement of an h-index of 67 (Web of Science) with 8 of these publications accumulating over 200 citations. This volume is dedicated to Professor Chris Nordin in recognition of his immense contribution to osteoporosis research and its prevention.
